# Epidemiologic Determinants for Modeling Pneumonic Plague Outbreaks

**DOI:** 10.3201/eid1004.030509

**Published:** 2004-04

**Authors:** Raymond Gani, Steve Leach

**Affiliations:** * Centre for Applied Microbiology & Research, Wilts, United Kingdom

**Keywords:** *Yersinia pestis*, pneumonic plague, mathematical modeling

## Abstract

Pneumonic plague poses a potentially increasing risk to humans in plague nonendemic regions either as a consequence of an aerosolized release or through importation of the disease. Pneumonic plague is person-to-person transmissible. We provide a quantitative assessment of transmissibility based on past outbreaks that shows that the average number of secondary cases per primary case (*R_0_*) was 1.3 (variance = 3.1), assuming a geometric probability distribution, prior to outbreak control measures. We also show that the latent and infectious periods can be approximated by using lognormal distributions with means (SD) of 4.3 (1.8) and 2.5 (1.2) days. Based on this parameter estimation, we construct a Markov-chain epidemic model to demonstrate the potential impact of delays in implementing outbreak control measures and increasing numbers of index cases on the incidence of cases in simulated outbreaks.

*Yersinia pestis* causes an enzootic vector-borne disease infecting rodents and fleas; humans can also become infected when exposed to zoonotic reservoirs. Infection in humans usually occurs in the form of bubonic plague when fleas that have previously fed on plague-infected rodents bite them. Secondary pneumonic plague may then occur if infection spreads to the lungs. Persons with secondary pneumonic plague become infectious and can transmit the disease to other persons by the respiratory route, causing primary pneumonic plague (*1*,*2*). Primary pneumonic plague is also person-to-person transmissible and can sustain cycles of human transmission independent of flea and rodent vectors. Bubonic plague can usually be treated successfully with antibmicrobials; however, secondary pneumonic plague and primary pneumonic plague require prompt antimicrobial treatment. Symptoms develop rapidly and are usually fatal (*1*,*3*,*4*). The recent discovery of antibiotic-resistant strains of *Y*. *pestis* (*5*) poses potential new concerns for therapeutic and prophylactic treatments during outbreaks.

The risk of importing *Y. pestis* to nonendemic regions may have increased over recent years. The worldwide extent of plague endemic-areas and the global incidence of reported disease have both increased (*6*), as have the volume and rapidity of national and international trade and travel. These factors raise the likelihood of importation either through travelers incubating plague (as occurred in New York 2002 [*7*]), or through importation of infected vectors, such as fleas or rats. Imported vectors then have the potential to initiate outbreaks of pneumonic plague.

Plague is also recognized as a potential weapon for bioterrorists (*3*,*8*–*11*) and has been used, or considered for use, as a biologic weapon in the past. From the 14th to the 18th century in Europe, attempts were made to spread plague in besieged cities by catapulting plague victims over the walls (*12*). During the 1930s, the Japanese military attempted to spread plague in China by dropping plague-infected fleas from aircraft (*12*). As late as the 1990s, the Union of Soviet Socialist Republics was developing plague as an aerosol agent to cause primary pneumonic plague in target populations (*9*). Recent training exercises in the United States have been conducted to test the abilities of healthcare systems to cope with large-scale aerosolized releases of *Y. pestis* into urban populations (*13*,*14*).

Given that primary pneumonic plague is transmissible person-to-person and outbreaks could occur as a consequence of importation or bioterrorism, it is essential to develop quantitative assessments of the transmissibility and kinetics of the disease that are as robust as possible to aid public health planning, including training exercises such as those referred to above. Without preparation, inappropriate responses such as those seen during the suspected outbreak of plague in Surat, India (1994), are inevitable; the tourist industry suffered, exports were affected, and excessive demands were placed upon healthcare systems. The losses in this case have been estimated to run into billions of U.S. dollars (*15*).

While there has been much discussion concerning the transmissibility of primary pneumonic plague, no quantitative estimates could be found in published literature. The qualitative assessments that were found varied considerably: some reports suggest that primary pneumonic plague is highly transmissible and infectious (*1*,*16*–*19*), while others suggest that it is not (*20*,*21*) or that intimate contact between persons is required for transmission (*22*,*23*).

Using mathematical models based on historic data, we quantitatively assess the transmissibility and potential health effects of primary pneumonic plague outbreaks under a range of assumptions. In this initial analysis, we consider only the immediate health effects due to primary pneumonic plague and not the possible long-term effects due to potentially establishing the pathogen in rodent reservoirs and subsequent risks for bubonic plague. Based on available epidemiologic evidence, the modeling assumes that persons, once infected, experience a nonsymptomatic latent period followed by a symptomatic infectious period during which they can transmit primary pneumonic plague to other persons. Thereafter, if infected persons are untreated they will die. The reported case-fatality rate is close to 100% (*1*,*3*,*4*).

To estimate the duration of the latent period and the infectious period, and the probability of transmission of primary pneumonic plague, data describing cases and transmission events were sought from well-documented outbreaks. Reports of sufficiently well-documented outbreaks were rare, and each of the outbreaks resulted in relatively small numbers of new cases of primary pneumonic plague. Since therapy may affect the duration of individual latent periods and infectious periods, only the data in reports from person who had not received therapy was used in this analysis for latent periods (*24*–*29*), and for infectious periods (*24*,*25*,*27*,*28*). Lognormal distributions were fitted to these data by maximizing the log-likelihood function. In subsequent modeling, the duration of individual latent periods and infectious periods could then be taken from the fitted lognormal distributions in Figure 1 with means (SD) of 4.3 (1.8) and 2.5 (1.2) days.

**Figure 1:**
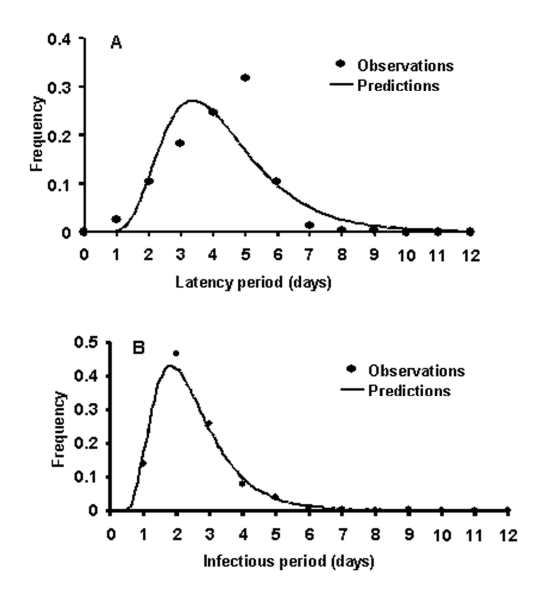
A. Frequency distribution for the latent period with a fitted lognormal distribution (n=224); B. frequency distribution of the length of the infectious period with a fitted lognormal distribution (n=225).

To estimate the transmission rate of primary pneumonic plague, only those transmission events from reports where the infecting persons could be unambiguously identified and where the infections had occurred before public health intervention were included in the analysis. The average number of infections generated by each infected person was then determined for each of the outbreaks documented in the Table, which varied from 0.8 to 3.0 (this variation most likely reflects the stochasticity that is inherent in very small outbreaks—see also discussion below). To obtain a stronger and more generalized estimate of transmissibility across all of the outbreaks, probability density functions (e.g., Poisson, geometric), were fitted to these data by maximizing the log-likelihood function for the probability and frequency of individual transmission events aggregated across the datasets. The geometric distribution gave the best fit to the data (*f(x) = p(*1*-p)^x^*, where *x* = no. secondary cases per primary case, *f(x)* = frequency and *p* = 0.43), and predicted an average of 1.3 secondary cases per primary case (R_0_) with variance of 3.1. This provides a probability density function (Figure 2) which was used in subsequent modeling to calculate the expected number of secondary cases per primary case for each person infected with primary pneumonic plague.

**Table:** Documented outbreaks of primary pneumonic plague from which transmission data were derived.

Y and location	Total of PP cases in outbreak^a^	No. of PP cases before intervention^b^	Transmission events	Average of secondary transmissions per primary transmission
Seattle USA, 1907 (30)	5	5	4	0.8
Oakland USA, 1919 (24)	13	6	12	2.0
Ecuador, 1939 (23)	18	4	6	1.5
Mukden, China, 1946 (25)	39	9	8	0.9
Rangoon, 1946 (31)	16	11	22	2.0
NW Madagascar, 1957 (32)	42	35	39	1.1
Zambia, 1993 (33)	3	3	2	0.7
Madagascar, 1997 (26)	18	1	3	3.0

^a^Includes index case. ^b^Only includes cases where the infecting person could be identified.

**Figure 2:**
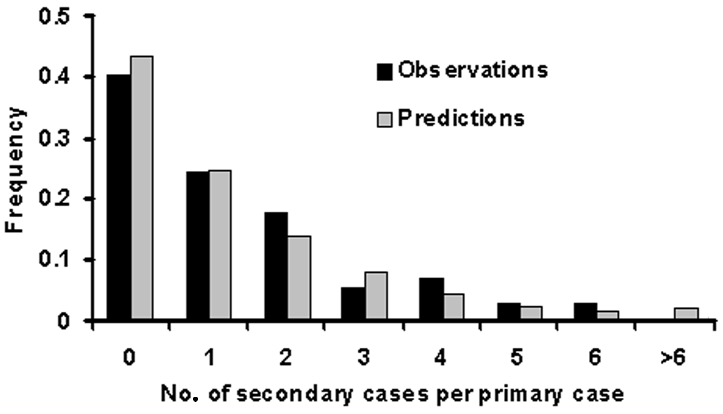
Frequency distributions for the number of secondary cases per primary case of primary pneumonic plague. Observations from outbreaks in Table are in black and the fitted geometric distribution in gray.

Documented 20th century outbreaks of primary pneumonic plague were often rapidly contained once they came to the attention of public health authorities (Figure 3). Even in the pre-antimicrobial era when outbreaks were not specifically identified as plague (e.g., the outbreak in Oakland in 1919 [24] that was thought to be a deadly influenza), the isolation of ill persons and observation and isolation of contacts were sufficient to rapidly control the outbreak. Contact tracing and isolation tended to be immediately effective because patients were infectious for only a short time, were very ill and unlikely to go out into the community, and any subsequent infections tended to be in those already caring for the patient (Figure 4). Very rarely were there cases where a prior infectious contact could not be identified. In addition, modern antimicrobial prophylaxis, when given in the incubation period, is close to 100% effective for pneumonic plague, greatly reducing any prospects of transmission from infected, but not yet symptomatic, persons (*3*,*22*,*26*,*34*,*35*). The subsequent modeling therefore assumes that once an outbreak has been identified, further transmission will be stopped. It is further assumed that a cumulative number of deaths are likely to have occurred before an outbreak comes to the attention of public health authorities and appropriate interventions are put in place, denoted *D_0_*.

**Figure 3:**
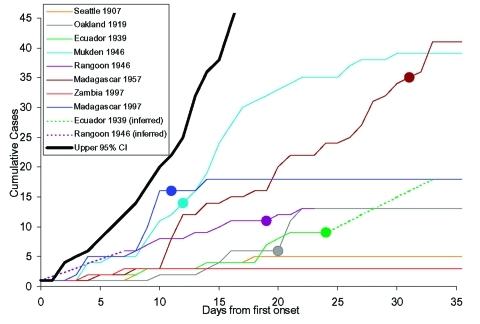
Epidemic curves for outbreaks in the Table and from the model. The curves plot cumulative cases at time of onset. Day 0 is the time of onset of index case, the circles represent the times at which disease control measures begin, those without circles ended without public health interventions. Dotted lines indicate missing data. The thicker yellow line represents the upper 95 percentile from the epidemic model, which rises roughly exponentially to a value of 256 by day 35.

**Figure 4:**
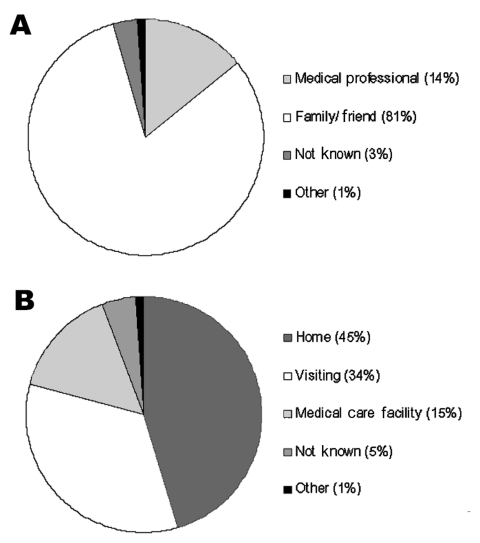
Distributions for the contexts of the transmission events for PPP by (A) type of contact with infectious individual (n=91), and (B) location of infectious contact when infected (n=86). Data aggregated from multiple sources (23–26,30–33), where these data were specified).

^A simple^ Markov-chain model was used to model disease outbreaks such that an individual *i* would have a latent period of *L_i_* and an infectious period of *I_i_*, where *L_i_* and *I_i_* were random deviates selected from the appropriate probability density functions in Figure 1. The individual *i* would then infect *T_i_* susceptible persons, where *T_i_* was a random deviate selected from the geometric probability density function described in Figure 2. As a simplifying assumption, new infections were assumed to occur within 1 day of *i* becoming infectious, as new infections were usually in close personal caregivers, few in number, and the symptomatic period of short duration. The upper 95th percentile from the multiple iterations of the model with no interventions applied is shown in Figure 3, along with the epidemic curves for each of the outbreaks listed in the Table. From the timings of the public health interventions that are shown in Figure 3, it is clear, ^with the exception of Mukden, 1946 (^*^25^*^), that^ the ^control measures were very effective in controlling all outbreaks; any subsequent cases occurred only as a result of infections incurred before the initiation of the control measures.^

After the introduction of latent infections into a community, infectious symptomatic cases will begin to appear over time. By the time an outbreak has been detected, there will potentially be a number of infectious persons in the community who can be estimated by using the modeling procedure described above. This number is critical in estimating the likely scale of response that might be required by public health authorities, giving a guide not only to the number of infectious people in the community at that point, but also an index for further onward transmission should responses be delayed. The model was thus used to numerically estimate a function, given by equation 1, that estimates the average number of infectious persons in the community with the potential to infect others, *I(t)*, at different times, *t*, following the initial introduction of different numbers of infection(s) (*N_0_*) into the population and prior to control measures being applied (i.e., prior to *D_0_* deaths having occurred).

*I*(*t*) = α*N_0_e*^β^*^t^* (equation 1)

where α = 0.3841 (SE = 0.00078) and β = 0.0734 (SE = 0.00005) for *t*≥5 days. The derived relationship does not hold well for *t*<5 days because of the delay until the onset of illness in the first cases. In addition, it may not hold for larger values of *N_0_* and *t* where nonlinear mixing patterns and depletion of susceptibles are likely to have an increasingly large effect on *I*(*t*). A different modeling strategy would probably be required to estimate the potential extent of outbreaks for much larger numbers of initial index cases, but such events are likely to be much less probable.

The transmission rate derived here for primary pneumonic plague is relatively low compared to many other communicable diseases (*36*), and in 43% of the simulated outbreaks initiated by one index case, no transmission occurred. However, the rapid onset of the infectious period (Figure 1) and the high variance associated with the transmission rate means that if control measures are not promptly and efficiently applied, in some instances much larger outbreaks could occur. For example, for those simulated outbreaks that did “take-off”, large numbers of cases could result before interventions halted further transmission (Figure 5). Small changes in *D_0_* considerably increased the probability of larger numbers of total expected cases (Figure 5A) and extended the lengths of outbreaks (Figure 5B).

**Figure 5:**
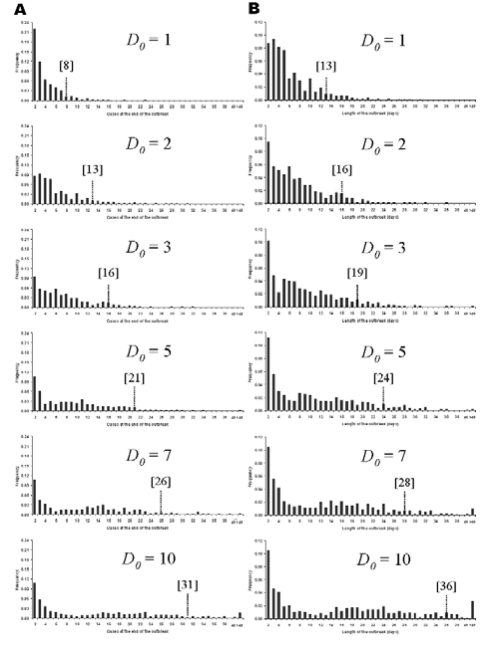
Frequency distributions for (A) the expected number of cases at the end of outbreaks, and (B) the expected lengths of outbreaks when different numbers of deaths are required to trigger public health interventions.

Where *N_0_* is large (e.g., following an efficient aerosolized release of *Y. pestis*), the dynamics associated with outbreaks will be considerably different than when *N_0_* is small for 2 key reasons. The first reason is that for large *N_0_*, the probability of transmission is more likely so that natural epidemic die-off will be an unlikely event. The second is that outbreak detection will occur more rapidly as it may not be necessary for multiple generations to have occurred before *D_0_* is reached. Thus, the changes in total numbers of cases per outbreak due to the variation in *D_0_* are relatively smaller when *N_0_* is higher (*c.f.* Figures 5 & 6, and panels in Figure 7) because the difference in the time to *D_0_* occurring become less as *N_0_* increases. Thus, for higher *N_0_*, *D_0_* becomes a less significant factor in determining the total number of cases per outbreak. However, for large *N_0_*, other factors are likely to impact on the control measures, such as limitations in the capacity of healthcare facilities and antimicrobial prophylaxis to cope with large numbers of cases. For large *N_0_* and larger ensuing outbreaks that might exceed response capacities, the assumption in the modeling here that transmission would be reduced effectively to zero following outbreak detection would have to be reconsidered in the light of resource constraints.

**Figure 6:**
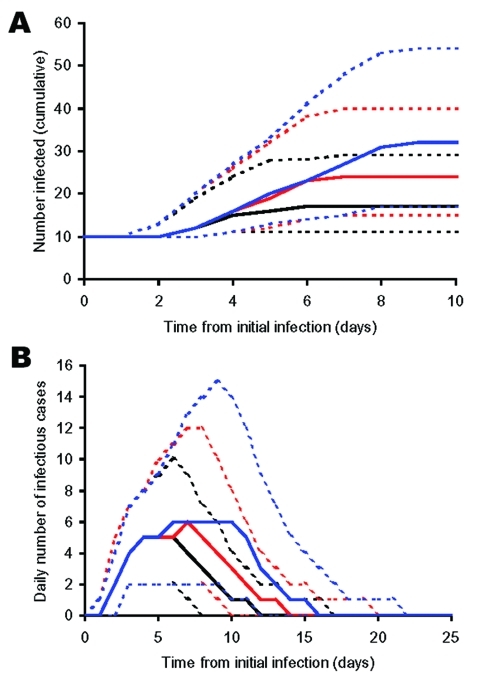
Estimates for (A) the cumulative number of people infected from the time of the first infection, and (B) daily number of infected people, where D_0_ = 1 (black), 5 (red) and 10 (blue). Solid lines represent the median number of cases from multiple iterations (n = 1000) of the model and the dotted lines give the upper and lower 95 percentiles.

**Figure 7:**
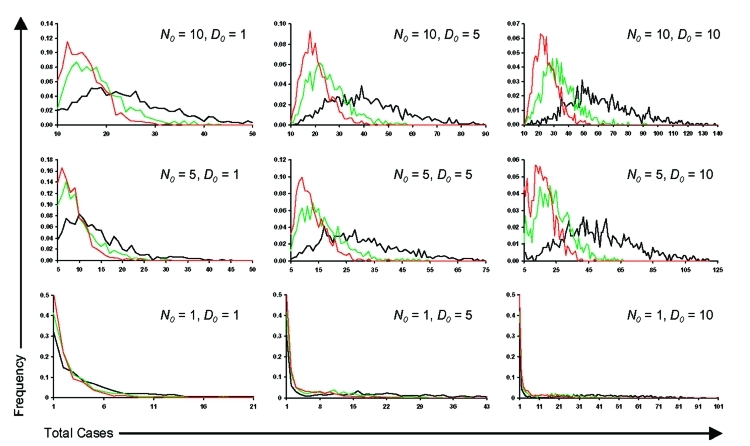
Variation in the expected number of cases at the end of an outbreak when N_0_, D_0,_ and R_0_ are varied across multiple iterations (n = 27,000) of the model (red denotes R_0_ = 0.96, green denotes R_0_ = 1.3, and black denotes R_0_ = 2.3). (N.B. Note scale changes).

Reducing the average number of secondary cases per primary cases (*R*) below one is a key step in controlling outbreaks, as this means that the number of new cases declines in successive generations of infection. Since the value of *R_0_* for primary pneumonic plague is already close to one, the control of potential outbreaks in most cases should be relatively straightforward and undemanding, especially if started by relatively few initial index case-patients. However, given that the upper and lower 95% confidence limits for the estimate of *R_0_* (based on the significance of the χ^2^-values derived from minimizing the log-likelihood function) are 2.3 (variance = 7.8) and 0.96 (variance = 1.9), outbreaks with higher values of *R_0_* in this range could result with greater probability in considerably large outbreaks that would be increasingly difficult to control unless measures were implemented quickly and efficiently (Figure 7).

The fact that the estimated *R_0_* is close to one reflects the frequent qualitative observation (*23**–**26**,**30**–**31**,**33*) that those infected tend to be those directly caring for ill persons either at home or in a healthcare setting (Figure 4). Given the close contact that was required for transmission and that transmission actually occurred relatively infrequently, the predominating issue determining the variability of transmission between outbreaks is likely to have been stochasticity. This assertion is supported by the results of the simulations, which demonstrate a range of potential sizes and lengths for outbreaks even for individual mean R_0_ values (Figures 5 & 7). Although cultural and other factors, such as social and healthcare structures, may well have been different across the outbreaks that have been analyzed, in most cases these factors probably had a relatively minor impact. Although the transmission rate of primary pneumonic plague appears to have been consistently low across these better documented outbreaks, stochastic effects could still generate significant outbreaks by chance (Figures 5 & 7), which coupled with the rapid kinetics of the infection means that such outbreaks could also develop rapidly. In the sensitivity analysis here, however, even such larger outbreaks rarely exceeded more than a hundred cases, even for the higher estimates of *R_0_*, *N_0,_* and *D_0_*. Of course, this assumes relatively small numbers of initial index cases (~*N_0_* < 10), relatively sensitive outbreak detection systems (~*D_0_* < 10), and prompt and efficient public health interventions (*R* tends to zero immediately following outbreak detection). Thus, the key element in the control of smaller outbreaks of primary pneumonic plague would be the acuity of disease surveillance systems and quick detection of outbreaks, the efficiency of which might depend significantly on the number of persons initially infected.
